# Professional Weaknesses

**Published:** 1856-06

**Authors:** 


### PROFESSIONAL WEAKNESSES.

BY A. A. B.

No profession can possibly hold a respectable position in the world, whose individual members fail to respect each other, or who disregard the high degree of honor which should ever be found to characterize their intercourse.

We have been induced to think of this subject from having become acquainted with a victim, by the want of courtesy, indeed it may be said of honesty, in the conduct of some members of our profession towards him, and to whom I would address a few remarks.

In the first place, I would speak of the practice of slanderously talking of neighboring practitioners, either in relation to their ability as dentists, or as men.

With this a gentleman can have nothing to do; it cannot enter into any portion of his necessities, or interests; it can neither elevate him in the mind of his hearers, nor can it add one particle of ability to his capacity; it can attach no glory to his name, no credit to his reputation; cannot even relieve the bitterness of a bad nature, nor ease a heart sore and guilty. Therefore it is a deadening occupation, utterly beneath a calling whose aim is usefulness, whose aspirations are noble and exalted. Unbefiitting a profession that can boast of members beloved for great powers, great hearts, and a high order of intellectuality of such men as labor night and day for the promotion of our science, the elevation of our standard, for the whole good of our professional body.

The man who can listen to the detraction of a co-worker in our ranks, without a blush of indignation towards the perpetrator of the wrong, is, indeed a sorrowful spectacle of a mistaken and misguided man. One who will have to wrestle, in mighty strength with a monster who leaves a slimy track in the foot-prints of its victim; one whose career through life will be bowed down with an oppressive load, whose solitary path will be cheered by no friendly voice, whose future is like a dark and fearful cloud suspended before the star of existence.

But he who can slander another, with whom he has no acquaintance, merely from jealousy, is, indeed, a weak creature, claiming the pity and contempt of all: a dirty infection, ulcerating the vitals of his profession, loathed and shunned by all honorable members.

We are, unfortunately, subjected to a nuisance in many respects, that is fast leveling the practice of dentistry to a mere craft, which the other professions are not in the least infested with;—*that is a system of underbidding.*

Physicians have established prices for their practice throughout whole communities, regulated by local association. Dentists have all prices at all times.

The consequence is, that a fair price for skillful operations, is seldom obtained, only by an exception to the general rule. One operates for five dollars a filling, others for four, three, two, and so on down to fifty cents, each claiming to do the best, all managing to secure this infatuation in some of each community. This is still worse in the mechanical department, some constructing full sets for $200, others for $20, the low price operators kidnapping from the higher, so that patients, with few exceptions, are induced to go about *shopping* for operations as they do for wearing apparel; *small* dentists becoming Jews in good earnest, varying in price from a large sum to a mere song.

Here we have reached the battle ground. Men who are skillful, require an equivalent price for their art, those of inferior grade, regulate their prices accordingly, and cry *extortion* to the more gifted man; for, of course, every professional man knows, that there is as much difference in the *operations* of dentists as there is in school boy sketches with Raphael's master-piece, as wide a difference in workmanship as there is exhibited in a penny watch and in a Gurgensen's best chronometer. Yet to applicants for dental services, who cannot but imperfectly judge of these things, there is no important difference, as they are led to believe in most cases. That between Dr. Toodle and Mr. Wright, the only difference will be found in Dr. T.——, asking a fair living price for his services, and Mr. W——, commanding an extortion from a presumptive position "that perhaps the real, solid truth is there is no more preference to be had," than will be found in securing a cherry ripe and a cherry red.

True, persons often learn from sad experience, that they have been deceived, and that there is a material difference between good prices and good operations, and low prices and low operations. Justice is sorely cheated, for the very fact of these patients seeking better operators in the end, secures the inferior one from exposure which is richly due him, as no gentleman who is a good dentist, will be found wasting precious moments in idle corrections for inferior persons, or in commenting upon conduct he is compelled to blush for.

Every man, however gifted, will fail sometimes. It is unavoidable, our operations being at times purely speculative.

The early career of all men, however noble their aspirations, must be characterized by the style and power of the novitiate; the first years of practice, are those of intense toil and doubt, crowned by failure as often as success, until struggling labor asserts her indomitable influence, and we find the novice grown into the skillful dentist. But the result is this, these errors, useful in themselves, as they force improvement upon us, are turned, by the weak members of the profession, to their advantage, and used as everlasting proofs of great incompetency, for in their hands they never become things of the past, but always monsters of the present.

It is often asked, why this state of things is not improved? Why have we not more general societies and local associations?

The reason appears to reside in the facts, that there is so great a preference to become a lion, rather than a useful dentist, to rule in a circle whose limits circumscribe one's house and office, rather than serve in the ranks of a profession struggling to rise to its true position. The fear of losing some advantage by exchanging experiences, or of finding a level beneath the self-loved and self-constituted standard; a selfish satisfaction in finding a business sufficiently lucrative to supply individual wants, leading to a careless regard of a profession that generously offers a handsome support to all deserving industry; perhaps a fear that personal intercourse would force the abandonment of secret practices to the disadvantage of their neighbor, and to their own fancied emolument.

Should these surmises be correct, what unfortunate interpretations of self-interest, of that sphere of usefulness which should be the aim of every man, of the means to reach a great ultimate advantage, and which must greatly tend to stamp the significance of weakness upon the calling.

Elevate the profession and you infinitely advance yourself, add to its advantages, and you increase your own usefulness in a ten-fold degree, extend its application, and you will have found an inexhaustible El dorado.—*New York Dental Recorder.*

